# Supplementary material for: Transcription factor 7-like 2 single nucleotide polymorphisms rs290487 and rs290481 are associated with dyslipidemia in the Balinese population
Source: PeerJ. 2022 Mar 22;10:e13149. doi: 10.7717/peerj.13149 (PMC8953500; doi:10.7717/peerj.13149)
Supplement: Supplemental Information 1 [file peerj-10-13149-s001.docx]

Table S1. Primer sequences for ARMS-PCR.

| **SNP** | **Primer** | **Sequence (5' 🡪 3')** | **Length (bp)** | **Allele** | **Amplicon size (bp)** |
| --- | --- | --- | --- | --- | --- |
| rs290487 (C>T) | Inner Forward | CAACCCAGTACAAATCATGGTGACACAAT | 29 | T | 211 |
|  | Inner Reverse | GATCAAACACCTTTCTCATTTTCAATTTTTCG | 32 | C | 295 |
|  | Outer Forward | GAATTGGCTTTGTAAAGAGCAGATTGTTATTCCT | 34 | - | 467 |
|  | Outer Reverse | ATACAGTTTGTGTTTTCTCCTCTCATGCTGC | 31 | - |  |
|  | | | | | |
| rs290481 (C>T) | Inner Forward | ACACGTGTTTCCAGTTGGAGTGTTATCATCT | 31 | T | 280 |
|  | Inner Reverse | CATCTAAGGACCCTAGAATATTGTCAGAACCCTG | 34 | C | 372 |
|  | Outer Forward | CTTCTGACCCAGAAGGAACAGACAGACCCTG | 31 | - | 587 |
|  | Outer Reverse | CATGTAAGAAATAGGAATGAGAATTTCAGAGCCCT | 35 | - |  |
