# Supplementary material for: Transcription factor 7-like 2 single nucleotide polymorphisms rs290487 and rs290481 are associated with dyslipidemia in the Balinese population
Source: PeerJ. 2022 Mar 22;10:e13149. doi: 10.7717/peerj.13149 (PMC8953500; doi:10.7717/peerj.13149)
Supplement: Supplemental Information 2 — HWE, Hardy−Weinberg equilibrium; LD, linkage disequilibrium. The p value for HWE was calculated using Pearson’s chi-squared test. [file peerj-10-13149-s002.docx]

Table S2. Genotype and allele frequencies, Hardy−Weinberg equilibrium and linkage disequilibrium.

| **SNP** | **Genotype/Allele** | **Frequency** | **HWE *p*** | **Pairwise LD** | |
| --- | --- | --- | --- | --- | --- |
|  |  |  |  | ***D*’** | **R^2^** |
| rs290487 | CC | 0.19 | 0.444 | 0.90 | 0.72 |
|  | CT | 0.51 |  |  |  |
|  | TT | 0.30 |  |  |  |
|  | C allele | 0.44 |  |  |  |
|  | T allele | 0.56 |  |  |  |
|  | | | |  |  |
| rs290481 | CC | 0.22 | 0.800 |  |  |
|  | CT | 0.49 |  |  |  |
|  | TT | 0.28 |  |  |  |
|  | C allele | 0.47 |  |  |  |
|  | T allele | 0.53 |  |  |  |

HWE, Hardy−Weinberg equilibrium; LD, linkage disequilibrium. The *p* value for HWE was calculated using Pearson’s chi-squared test.
