# Supplementary material for: Transcription factor 7-like 2 single nucleotide polymorphisms rs290487 and rs290481 are associated with dyslipidemia in the Balinese population
Source: PeerJ. 2022 Mar 22;10:e13149. doi: 10.7717/peerj.13149 (PMC8953500; doi:10.7717/peerj.13149)
Supplement: Supplemental Information 3 — Abbreviations: TG, triglycerides; LDL-C, low-density lipoprotein cholesterol; HDL-C, high-density lipoprotein cholesterol; TC, total cholesterol. Dyslipidemia was defined by the presence of at least one of the following criteria: high TG (≥200 mg/dL), high LDL-C (≥160 mg/dL), low HDL-C (<40 mg/dL) or high TC (≥240 mg/dL) (NCEP, 2002). Comparisons of genetic distribution were performed using the Pearson’s chi-squared test. The significant Bonferroni’s corrected p values are in bold (p < 0.025). [file peerj-10-13149-s003.docx]

Table S3. Comparison of genotypic distribution between subjects who were affected and not affected with dyslipidemia.

| **Trait** | **Genotype** | **Frequency (rs290487)** | | | **Frequency (rs290481)** | | |
| --- | --- | --- | --- | --- | --- | --- | --- |
|  |  | **Non-Affected Subjects** | **Affected Subjects** | ***p*** | **Non-Affected Subjects** | **Affected Subjects** | ***p*** |
| Dyslipidemia | CC | 0.17 | 0.22 | 0.067 | 0.21 | 0.25 | **0.016** |
|  | CT | 0.49 | 0.54 |  | 0.47 | 0.54 |  |
|  | TT | 0.34 | 0.25 |  | 0.33 | 0.21 |  |
|  | | | | | | | |
| High TG | CC | 0.19 | 0.17 | 0.222 | 0.23 | 0.21 | 0.078 |
|  | CT | 0.49 | 0.59 |  | 0.47 | 0.59 |  |
|  | TT | 0.32 | 0.24 |  | 0.30 | 0.20 |  |
|  | | | | | | | |
| High LDL-C | CC | 0.18 | 0.23 | 0.041 | 0.22 | 0.26 | 0.089 |
|  | CT | 0.50 | 0.60 |  | 0.48 | 0.57 |  |
|  | TT | 0.32 | 0.17 |  | 0.30 | 0.17 |  |
|  | | | | | | | |
| Low HDL-C | CC | 0.19 | 0.18 | 0.916 | 0.22 | 0.22 | 0.792 |
|  | CT | 0.51 | 0.50 |  | 0.49 | 0.53 |  |
|  | TT | 0.30 | 0.34 |  | 0.29 | 0.26 |  |
|  | | | | | | | |
| High TC | CC | 0.17 | 0.26 | **0.003** | 0.21 | 0.29 | **0.009** |
|  | CT | 0.50 | 0.60 |  | 0.48 | 0.56 |  |
|  | TT | 0.33 | 0.15 |  | 0.31 | 0.15 |  |

Abbreviations: TG, triglycerides; LDL-C, low-density lipoprotein cholesterol; HDL-C, high-density lipoprotein cholesterol; TC, total cholesterol. Dyslipidemia was defined by the presence of at least one of the following criteria: high TG (≥200 mg/dL), high LDL-C (≥160 mg/dL), low HDL-C (<40 mg/dL) or high TC (≥240 mg/dL) (NCEP, 2002). Comparisons of genetic distribution were performed using the Pearson’s chi-squared test. The significant Bonferroni’s corrected *p* values are in bold (*p*<0.025).
