# Supplementary material for: Transcription factor 7-like 2 single nucleotide polymorphisms rs290487 and rs290481 are associated with dyslipidemia in the Balinese population
Source: PeerJ. 2022 Mar 22;10:e13149. doi: 10.7717/peerj.13149 (PMC8953500; doi:10.7717/peerj.13149)
Supplement: Supplemental Information 4 — Abbreviations: OR, odds ratio; 95%CI, 95% confidence interval. Dyslipidemia was defined by the presence of one of the following criteria: high TG (≥200 mg/dL), high LDL-C (≥160 mg/dL), low HDL-C (<40 mg/dL) or high TC (≥240 mg/dL) (NCEP, 2002). Interaction analyses were performed using the likelihood ratio test. The significant interaction p values are in bold (p < 0.100). [file peerj-10-13149-s004.docx]

| **Genetic Model** | **Genotype** | **Dyslipidemia Risk** | | | |
| --- | --- | --- | --- | --- | --- |
|  |  | **rs290487 × Obesity** | | **rs290481 × Obesity** | |
|  |  | **OR (95% CI)** | ***p_interaction_*** | **OR (95% CI)** | ***p_interaction_*** |
| Additive | TT | Reference |  | Reference |  |
|  | TC | 2.58 (1.08-6.27) | **0.034** | 3.22 (1.29-8.16) | **0.013** |
|  | CC | 0.76 (0.25-2.26) | 0.622 | 0.71 (0.24-2.07) | 0.529 |
| Dominant | TT | Reference |  | Reference |  |
|  | TC+CC | 1.84 (0.81-4.22) | 0.148 | 1.96 (0.83-4.67) | 0.121 |
| Recessive | TT+TC | Reference |  | Reference |  |
|  | CC | 0.41 (0.16-1.05) | **0.064** | 0.33 (0.13-0.81) | **0.015** |

Table S4. The interaction between rs290487 and rs290481 SNPs and obesity status on dyslipidemia risk under all genetic models.

Abbreviations: OR, odds ratio; 95%CI, 95% confidence interval. Dyslipidemia was defined by the presence of one of the following criteria: high TG (≥200 mg/dL), high LDL-C (≥160 mg/dL), low HDL-C (<40 mg/dL) or high TC (≥240 mg/dL) (NCEP, 2002). Interaction analyses were performed using the likelihood ratio test. The significant interaction *p* values are in bold (*p* <0.100).
