# Supplementary material for: Transcription factor 7-like 2 single nucleotide polymorphisms rs290487 and rs290481 are associated with dyslipidemia in the Balinese population
Source: PeerJ. 2022 Mar 22;10:e13149. doi: 10.7717/peerj.13149 (PMC8953500; doi:10.7717/peerj.13149)
Supplement: Supplemental Information 5 — Abbreviations: OR, odds ratio; 95%CI, 95% confidence interval. High TyG index (≥8.85). The optimal cutoff point for a high TyG index (8.85) for dyslipidemia was analyzed using the Youden index in “OptimalCutpoints” package (Lopez-Raton & Xose Rodriguez-Alvarez, 2021). Association analysis was performed using likelihood ratio test, adjusted by age, sex, population, and obesity (BMI ≥ 25 kg/m2 (WHO, 2000)) [file peerj-10-13149-s005.docx]

Table S5. The associations between rs290487 and rs290481 with TyG Index.

| **Trait** | **SNPs** | **Genetic Model** | **OR (95%CI)** | ***p*** |
| --- | --- | --- | --- | --- |
| High TyG Index | rs290487 | Additive | 1.01 (0.77-1.33) | 0.934 |
|  |  | Dominant | 1.13 (0.75-1.72) | 0.540 |
|  |  | Recessive | 0.86 (0.53-1.40) | 0.565 |
|  | rs290481 | Additive | 1.08 (0.83-1.41) | 0.546 |
|  |  | Dominant | 1.16 (0.77-1.77) | 0.464 |
|  |  | Recessive | 1.05 (0.67-1.64) | 0.817 |

Abbreviations: OR, odds ratio; 95%CI, 95% confidence interval. High TyG index (≥8.85). The optimal cutoff point for a high TyG index (8.85) for dyslipidemia was analyzed using the Youden index in “OptimalCutpoints” package (Lopez-Raton & Xose Rodriguez-Alvarez, 2021). Association analysis was performed using likelihood ratio test, adjusted by age, sex, population, and obesity (BMI ≥25 kg/m^2^ (WHO, 2000))
